# Effect of Intraperitoneal ^224^Radium-Labelled Microparticles on Compartmentalized Inflammation After Cytoreductive Surgery and Hypertherm Intraperitoneal Chemotherapy

**DOI:** 10.1177/15330338231192902

**Published:** 2023-08-14

**Authors:** Ebbe Billmann Thorgersen, Jørund Asvall, Camilla Schjalm, Karin Ekholt McAdam, Øyvind Sverre Bruland, Stein Gunnar Larsen, Tom Eirik Mollnes

**Affiliations:** 1 Department of Gastroenterological Surgery, 155272 Oslo University Hospital, The Radium Hospital, Oslo, Norway; 2 Department of Research and Development, Division of Emergencies and Critical Care, 155272 Oslo University Hospital, Oslo, Norway; 3 Institute of Clinical Medicine, 6305 University of Oslo, Oslo, Norway; 4 Department of Immunology, Oslo University Hospital Rikshospitalet, Oslo, Norway; 5 Department of Oncology, 155272 Oslo University Hospital, The Radium Hospital, Oslo, Norway; 6 Research Laboratory, 376869 Nordland Hospital, Bodø, Norway

**Keywords:** inflammation, colorectal cancer, peritoneal metastasis, cytoreductive surgery, hyperthermic intraperitoneal chemotherapy HIPEC, α-emitting radionuclide therapy

## Abstract

Despite extensive treatment with surgery and chemotherapy many patients with peritoneal metastases from colorectal cancer experience intraperitoneal disease relapse. The α-emitting ^224^radium-labelled microparticle radionuclide therapeutic Radspherin® is being explored as a novel treatment option for these patients. Radspherin® is specially designed to give local radiation to the surface of the peritoneal cavity and potentially kill remaining attached micrometastases as well as free-floating cancer cells, thus preventing future relapse. The effect of Radspherin® on the immune system is not known. Systemic and local inflammatory responses were analyzed in plasma, intraperitoneal fluid and urine collected prospectively as part of a phase 1 dose-escalation study of intraperitoneal instillation of the α-emitting therapeutic radiopharmaceutical Radspherin®, at baseline and the first 7 postoperative days from nine patients undergoing cytoreductive surgery and hyperthermic intraperitoneal chemotherapy. All patients additionally received intraperitoneal instillation of Radspherin® on postoperative day 2. Complement activation products C3bc and the terminal complement complex were analyzed using enzyme-linked immunosorbent assay. Cytokines (*n* = 27), including interleukins, chemokines, interferons and growth factors, were analyzed using multiplex technique. The time course and magnitude of the postoperative cytokine response after cytoreductive surgery and hyperthermic intraperitoneal chemotherapy displayed a modest systemic response in plasma, in contrast to a substantial local intraperitoneal response. After administration of Radspherin®, a significant increase (*P* < 0.05) in TNF and MIP-1β was observed in both plasma and peritoneal fluid, whereas IL-9 increased only in plasma and IFNγ and IL1-RA only in peritoneal fluid. Only minor changes were seen for the majority of the inflammatory markers after Radspherin® administration. Our study showed a predominately local rather than systemic inflammatory response to cytoreductive surgery and hyperthermic intraperitoneal chemotherapy. Radspherin® had overall modest impact on the inflammation.

## Introduction

Cytoreductive surgery (CRS) and hyperthermic intraperitoneal chemotherapy (HIPEC) is established as treatment for peritoneal metastasis from colorectal cancer (PM-CRC) in selected patients with resectable disease.^1^ CRS–HIPEC involves resection of all visible tumor tissue within the peritoneal cavity, with subsequent perfusion of the peritoneal cavity with heated chemotherapy.^2^ Despite this extensive treatment, many patients experience intraperitoneal disease relapse, and novel treatment options are highly desired.^3^

Radium-224 (^224^Ra) adsorbed in calcium carbonate microparticles (^224^Ra-CaCO_3_-MP) (Radspherin®) is a novel treatment option explored as an adjunct to CRS–HIPEC for PM-CRC.^4^ Short-range α-particle radiation, distributed throughout the peritoneal cavity, could potentially kill residual cancer cells, free- floating or attached to the serosal surfaces.^4,5^

Little is known of the effect on the immune system of the complex and lengthy surgery of CRS–HIPEC neither locally nor systemically. Recent results point to a substantial local intraperitoneal inflammation early postoperatively the first 2 days after surgery, with less pronounced effect systemically.^6^ Furthermore, the immunological impact of this procedure in urine, reflecting both local and systemic activation, is not known.

The aim of the present study was to explore the inflammatory response the first week after CRS–HIPEC for PM-CRC and the impact of additional intraperitoneal (IP) treatment with Radspherin® at the recommended activity dose of 7 MBq,^4^ administered on postoperative day 2.

## Material and Methods

### Patients and Treatments

A first-in-human, prospective phase 1 dose-escalation study of intraperitoneal instillation of the α-emitting therapeutic radiopharmaceutical Radspherin® was conducted at two specialized CRS–HIPEC centers in Oslo, Norway and Uppsala, Sweden.^4^ An investigator initiated immunological arm linked to this study was conducted in the Norwegian patient cohort. All patients had suspected PM from histologically verified CRC and were selected for CRS–HIPEC. CRS was performed with the intention to remove all macroscopically visible tumors. All study patients underwent treatment with complete CRS and standard mitomycin C (MMC)-based HIPEC; 35 mg/m^2^ (maximum 70 mg), administered for 90 minutes in three fractions at 41.5 °C using “a closed technique with open abdomen.” Briefly, after CRS (“open abdomen”) before the HIPEC procedure, a frame and a plastic wrap was mounted and adapted to the surgical field (“closed technique”) as a measure to protect operating theatre personnel from chemotherapy exposure.^7^ Radspherin® was injected in the abdominal cavity through a catheter when the patients had stabilized after CRS–HIPEC, 2 days after the procedure. The phase 1 study was organized in four phases; a dose escalation with a 3 + 3 design with the patients given escalating doses starting at 1 MBq followed by 2, 4, and 7 MBq. No dose-limiting toxicity was observed.^4^ The study also involved an expansion cohort with six subjects at the highest safe activity-dose. The nine patients (three in the dose escalating part of the protocol and six in the expansion cohort, the latter also studying dosimetry) that received the highest dose in the study (7 MBq) were included in the immunological arm of the study.

Dose-calibrated Radspherin® (up to 10 mL containing 0.7-1 g of particles) was prepared at the Nuclear Medicine Department at site and administered as a single bolus injection via a three-way luer-lock connected to the inserted peritoneal catheter on postoperative day 2. After the injection the catheter and syringes were flushed with about 250 mL of isotonic solution, all drains were kept clamped for a minimum of 72 hours, except briefly reopened for the peritoneal fluid (PF) study sampling. For additional study details, see Larsen et al.^4^

The Accordion Severity Grading System of Surgical Complications was used to score surgical complications within the first 30 days (Table 1).^8^

**TABLE 1.** Patient Characteristics.

	*n* = 9
Gender	
Female	7 (78)^a^
Male	2 (22)
Age (years)	60 (28-68)^b^
BMI^c^	26 (21-32)
ECOG^d^
ECOG 0	8 (89)
ECOG 1	1 (11)
ASA^e^
ASA 1-2	8 (89)
ASA 3-4	1 (11)
Diagnosis
PM-CRC^f^	9 (100)
CRP screening (mg/L)	2 (1-58)
CEA (mcg/L)	2 (2-121)
CA 125 (U/mL)	31 (11-97)
CA 19-9 (U/mL)	14 (5-97)
Peritoneal cancer index (PCI)	8 (3-17)
Operating time (minutes)	410 (305-509)
Peroperative blood loss (mL)	400 (50-600)
Peroperative transfusion
RBC (units)	0 (0-2)
Plasma (units)	-
Platelets (units)	-
Length of stay (days)	11 (9-16)
Accordion^g^
0-1	4 (44)
2	5 (56)

^a^ Number of patients and percentage.

^b^ Median and range.

^c^ Body mass index.

^d^ Eastern Cooperative Oncology Group performance status.

^e^ American Society of Anesthesiologists physical status classification system.

^f^ Peritoneal metastasis (from) colorectal cancer.

^g^ Accordion severity grading system (expanded) of surgical complications within the first 30 days.

### Sampling and Processing

All time points for bio-sampling were harmonized with the already approved schedule for radioactivity-measurements as part of dosimetry-part of the phase 1 study.

Blood samples were collected at baseline (prior to CRS–HIPEC) and daily postoperatively throughout the sixth postoperative day, except for the second postoperative day where two samples were collected, that is before and after instillation of Radspherin®. The samples were centrifuged at 3000×*g* for 10 minutes and the EDTA plasma was stored in aliquots at -80 °C until analysis.

PF was collected in the evening of the day of CRS–HIPEC and twice daily until the morning the seventh postoperative day, from either of the indwelling abdominal drains. The samples were centrifuged at 3000×*g* for 10 minutes, aliquoted to EDTA-containing microvials and stored at -80 °C until analysis.

Urine was collected at baseline (prior to CRS–HIPEC) and daily throughout the seventh postoperative day, except for the second postoperative day where two samples were collected, before and after instillation of the study medicine. Urine was aliquoted to EDTA-containing microvials and stored in aliquots at -80 °C until analysis.

### Total Protein Measurement

The total protein concentration in plasma samples, PF and urine was measured with Bio-Rad protein Assay (Bio-Rad Laboratories, Hercules, CA) and Bio-Rad Protein Assay Standard II (Bio-Rad Laboratories, Hercules, CA).

### Cytokine Analysis

Plasma, PF, and urine samples were thawed on slush ice and analyzed using a multiplex cytokine assay (Bio-Plex Human Cytokine 27-Plex Panel, Bio-Rad Laboratories Inc., Hercules, CA) containing the following cytokines, chemokines and growth factors: interleukin (IL) 1 beta (IL-1β), IL-1 receptor antagonist (IL-1Ra), IL-2, IL-4, IL-5, IL-6, IL-7, IL-8 (CXCL8), IL-9, IL-10, IL-12 p70, IL-13, IL-15, IL-17, eotaxin (CCL11), basic fibroblast growth factor (bFGF), granulocyte colony stimulating factor (G-CSF), granulocyte-macrophage colony stimulating factor (GM-CSF), interferon gamma (IFN-γ), interferon gamma-induced protein/chemokine (C-X-C motif) ligand 10 (IP-10 or CXCL10), monocyte chemotactic protein 1 (MCP-1 or CCL2), macrophage inflammatory protein-1-alpha (MIP-1α or CCL3), macrophage inflammatory protein-1-beta (MIP-1β or CCL4), platelet-derived growth factor-BB (PDGF-BB), regulated upon activation T cell expressed and secreted (RANTES or CCL5), tumor necrosis factor (TNF), and vascular endothelial growth factor (VEGF). The analysis was performed according to the instructions from the manufacturer.

### Complement Analysis

In-house enzyme-linked immunosorbent assays (ELISA) were used to measure C3bc and fluid-phase C5b-9 (TCC) concentrations in EDTA plasma, PF and urine samples, as described in detail previously,^9,10^ and later slightly modified as detailed.^11^

### Data Presentation and Statistical Analysis

Clinicopathological data are presented as median and range or percentage of the study population. The complement and cytokine data were presented as mean and standard error of the mean (SEM). A paired *t*-test was used to compare the response before and after instillation of Radspherin® (time-point 2m and 2e, respectively). The samples before and after instillation of the study drug are marked with light grey circles in the figures. A sample size calculation was not conducted as it was out of scope for a study like this. The limited number of patients may influence the statistical significance. GraphPad Prism 7.02 (GraphPad Software, San Diego, CA) and SPSS software (version 25, IBM SPSS, Chicago, IL) were used for the analysis.

## Results

### Patient Characteristics

Of the patients included, 78% were females, and the median age was 60 years (Table 1). The median Peritoneal Cancer Index (PCI) was 8 with a range from 3 to 17. No major complications were detected in the time course that could influence the immunological results in the patients. All patients had an Accordion score equal or below 2 on a scale from 0 to 6. Central clinicopathological parameters are presented in Table 1.

### Total Protein Measurements

The total protein measurement displayed different time courses in the three compartments examined (Supplementary data, Figure S1). In plasma, there was a fall from the screening sample to the evening sample after CRS–HIPEC and thereafter a flat course. No effect of Radspherin® instillation was seen on the measurements. In PF, there was a gradual increase from the evening sample after CRS–HIPEC to the morning sample before instillation of Radspherin®. A decrease in concentration was seen after instillation of Radspherin®. In urine an increase in the amount of protein was seen after CRS–HIPEC until the morning sample before instillation of Radspherin®, thereafter a gradual fall to baseline levels was observed.

### Complement Analysis

**Figure 1.**
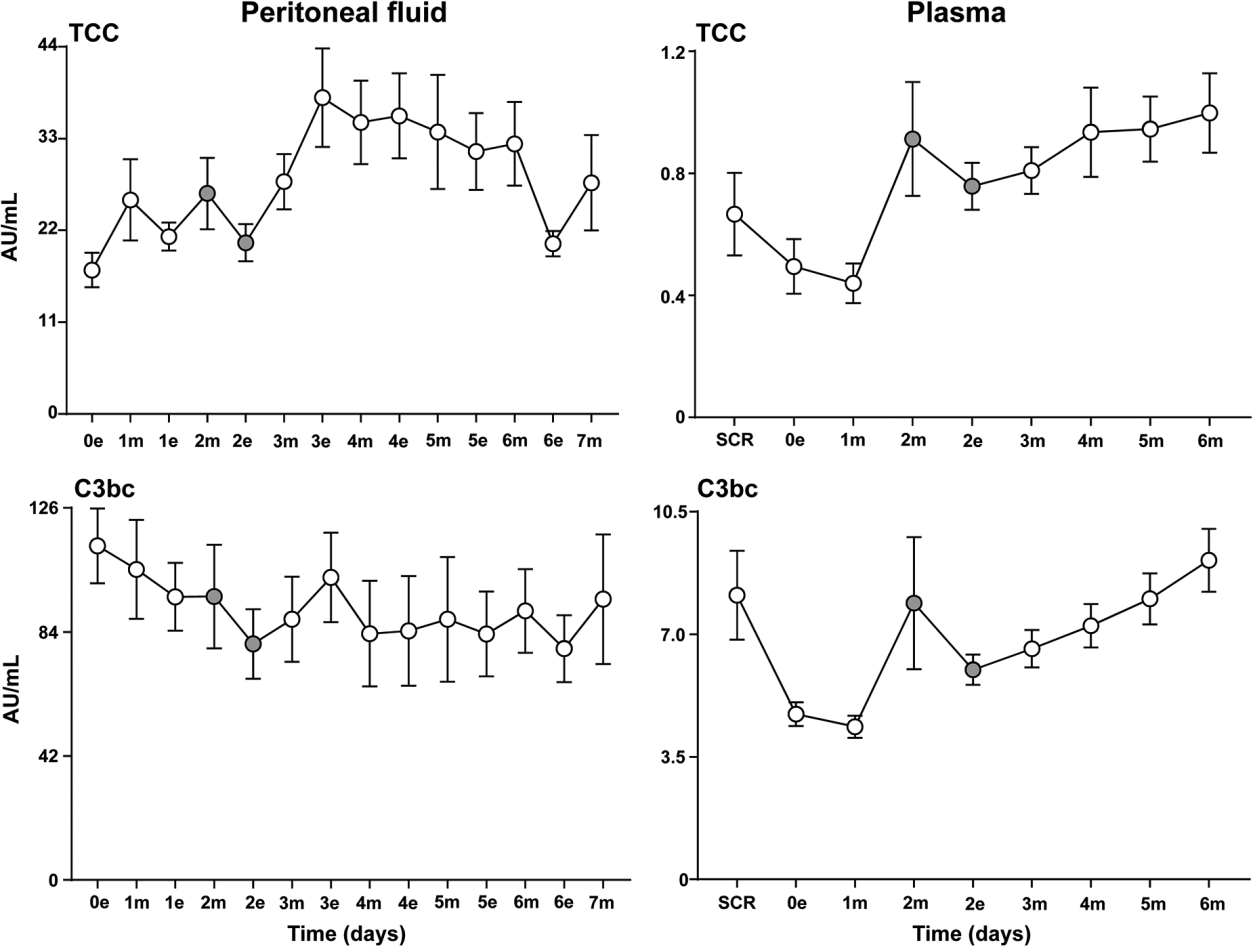
Complement activation in peritoneal fluid and plasma. Analysis of the terminal complement complex (TCC) and the central complement cascade product C3bc in peritoneal fluid and plasma samples on the evening the day of surgery (0e) and at baseline (SCR), respectively, and the following seven (in PF) and six (in plasma) postoperative days after CRS–HIPEC (*n* = 9). Intraperitoneal instillation of the α-emitting radionuclide therapeutic Radspherin® was conducted between time-point 2m and 2e (grey circles). Data are presented as mean and standard error of the mean. SCR = screening (baseline before surgery), 0 = day of surgery, 1-7 = postoperative days. m = morning sample. e = evening sample. Grey circles = time-points before and after instillation of Radspherin®.

In general, plasma levels of TCC and C3bc were 10 to 20 folds lower than corresponding intraperitoneal values (PF) (Figure 1). In plasma, TCC and C3bc revealed identical patterns of complement activation. A decrease from baseline (screening) to the morning sample the first postoperative day was seen, followed by an abrupt increase with a peak the next morning (the second postoperative day). A non-significant decrease after instillation of Radspherin® was seen, followed by a modest continuous increase during the next days.

In PF, a non-significant decrease after instillation of Radspherin® for both TCC and C3bc was seen, similar to the observations in plasma, followed by an activation of complement the following two time points.

No complement activation was detected in urine.

### Plasma Cytokines

Twelve of the 27 cytokines in the assay (IL-2, IL-4, IL-5, IL-7, IL-10, IL-12 p70, IL-13, IL-15, bFGF, GM-CSF, PDGF-BB and VEGF) were below the detection limit and were excluded from further analysis. In general, plasma values for the remaining 15 cytokines included were 2 to 35 folds lower than the corresponding intraperitoneal values (PF).

**Figure 2.**
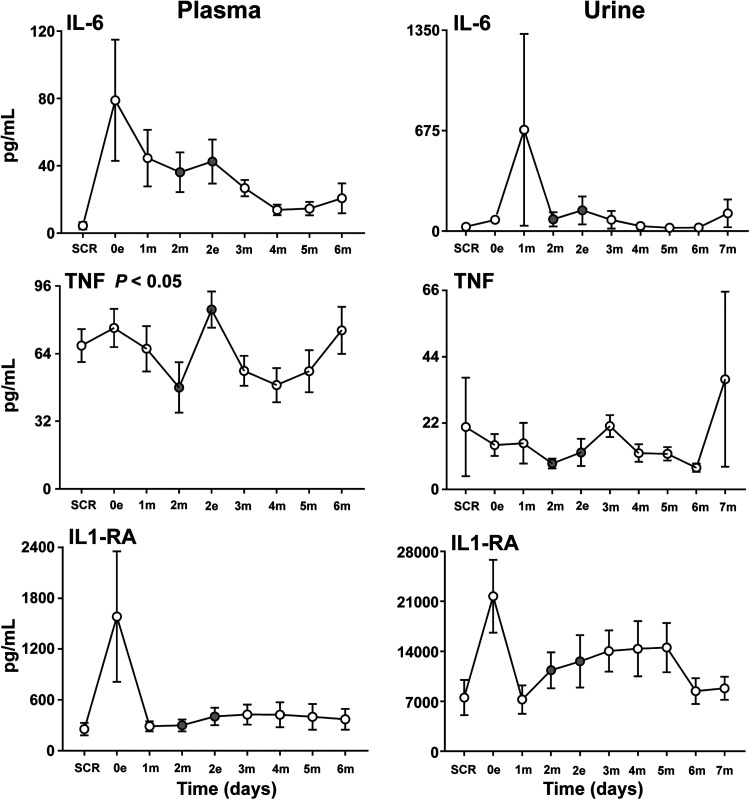
Cytokines in plasma and urine. Analysis of the proinflammatory cytokines IL-6 and TNF and the anti-inflammatory cytokine IL1-RA in plasma and urine samples at baseline (SCR), the day of surgery (0e) and the following six (in plasma) and seven (in urine) postoperative days after CRS–HIPEC (*n* = 9). Intraperitoneal instillation of the α-emitting radionuclide therapeutic Radspherin® was conducted between time-point 2m and 2e (grey circles). Data are presented as mean and standard error of the mean. SCR = screening (baseline before surgery), 0 = day of surgery, 1-7 = postoperative days. m = morning sample. e = evening sample. Grey circles = time-points before and after instillation of Radspherin®. *P*-values <0.05 were considered statistically significant.

The following five cytokines had a modest to steep increase from baseline to the first post-operative time-point: IL-6, IL1-RA (Figure 2), G-CSF (Figure S2), MCP-1 (Figure S3), and IL-8 (Figure S4). Three cytokines had a steep decrease from baseline to the first post-operative time-point: eotaxin (Figure S3), IL-8, and IP-10 (Figure S4), while the rest had modest changes from baseline.

Interestingly, three cytokines, TNF (Figure 2), IL-9 (Figure S2) and MIP-1β (Figure S3), increased significantly (*P* < 0.05) after IP instillation of Radspherin®. Four cytokines, IL-1β, IL-17 (both Figure S2), IL-8 (Figure S4) and MCP-1, decreased non-significantly after the instillation, while the rest had a non-significant increase or were unchanged. For some cytokines, in particular IL-1β, IL-17 and INFγ, the observed patterns and differences were difficult to interpret because of low overall values and values that was close to or just below the lower detection limit.

### Peritoneal Fluid Cytokines

In the peritoneal fluid 25 of the 27 cytokines measured were detected. PDGF-BB and VEGF were below the detection limit and were excluded from further analysis.

**Figure 3.**
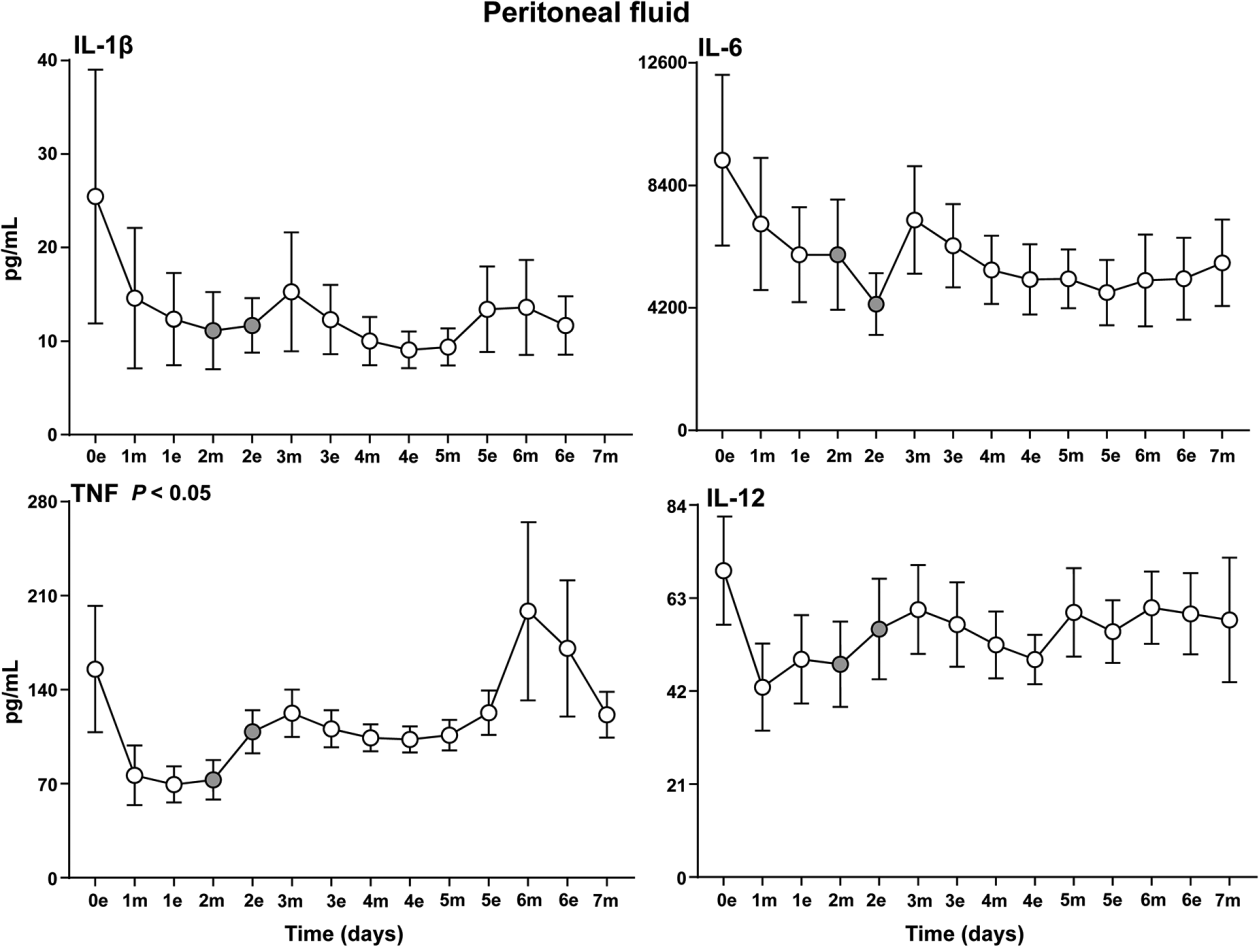
Proinflammatory cytokines in peritoneal fluid. Analysis of proinflammatory cytokines IL-1β, IL-6, TNF and IL-12 in peritoneal fluid on the evening the day of surgery (0e) and the following seven postoperative days after CRS–HIPEC (*n* = 9). Intraperitoneal instillation of the α-emitting radionuclide therapeutic Radspherin® was conducted between time-point 2m and 2e (grey circles). Data are presented as mean and standard error of the mean. SCR = screening (baseline before surgery), 0 = day of surgery, 1-7 = postoperative days. m = morning sample. e = evening sample. Grey circles = time-points before and after instillation of Radspherin®. *P*-values <0.05 were considered statistically significant.

**Figure 4.**
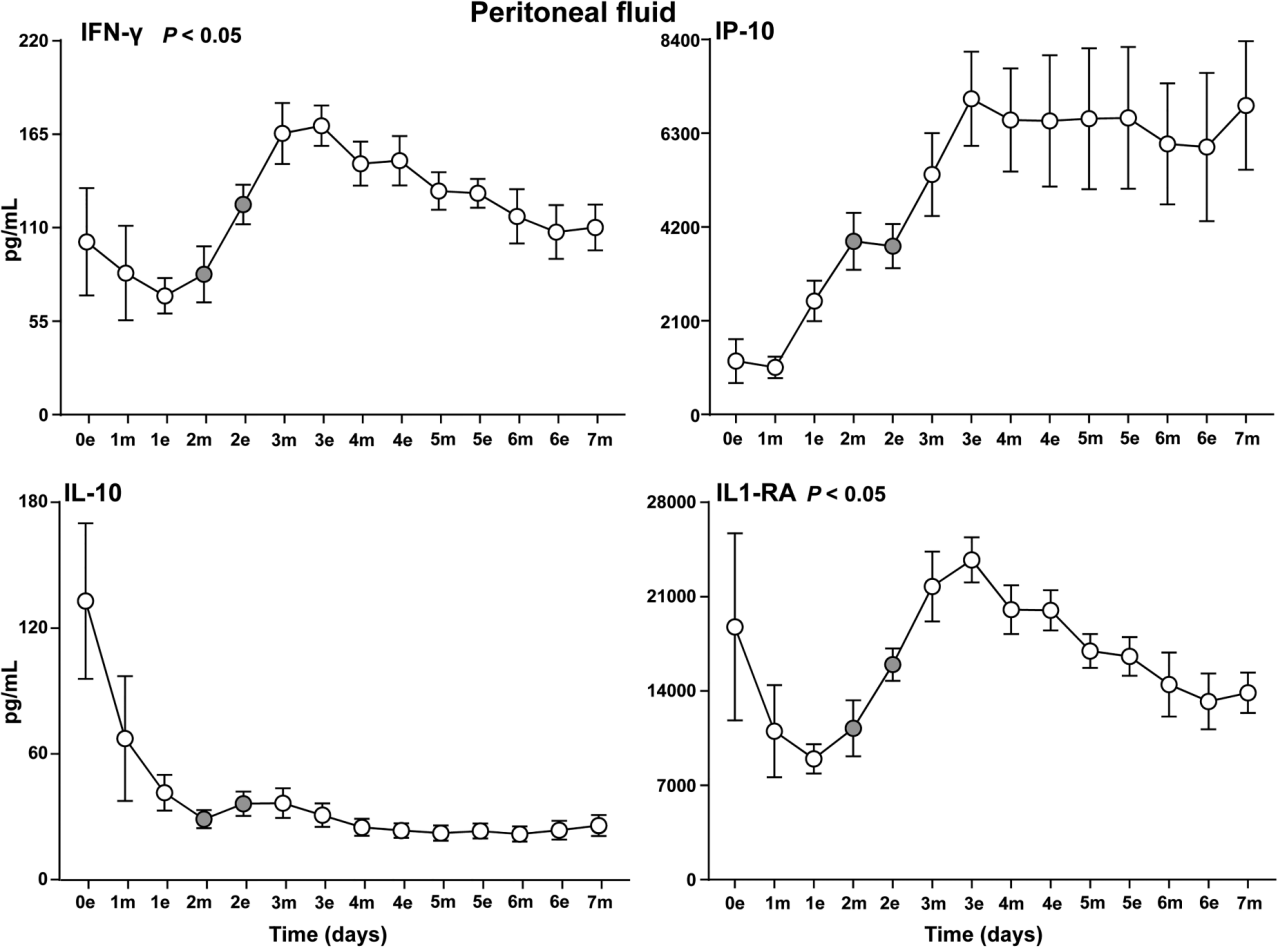
IFNγ, IP-10, IL-1RA and IL-10 in peritoneal fluid. Analysis of the cytokines IFN-γ and IP-10, IL-1RA and IL-10 in peritoneal fluid on the evening the day of surgery (0e) and the following seven postoperative days after CRS–HIPEC (*n* = 9). Intraperitoneal instillation of the α-emitting radionuclide therapeutic Radspherin® was conducted between time-point 2m and 2e (grey circles). Data are presented as mean and standard error of the mean. SCR = screening (baseline before surgery), 0 = day of operation, 1-7 = postoperative days. m = morning sample. e = evening sample. Grey circles = time-points before and after instillation of Radspherin®. *P*-values <0.05 were considered statistically significant.

**Figure 5.**
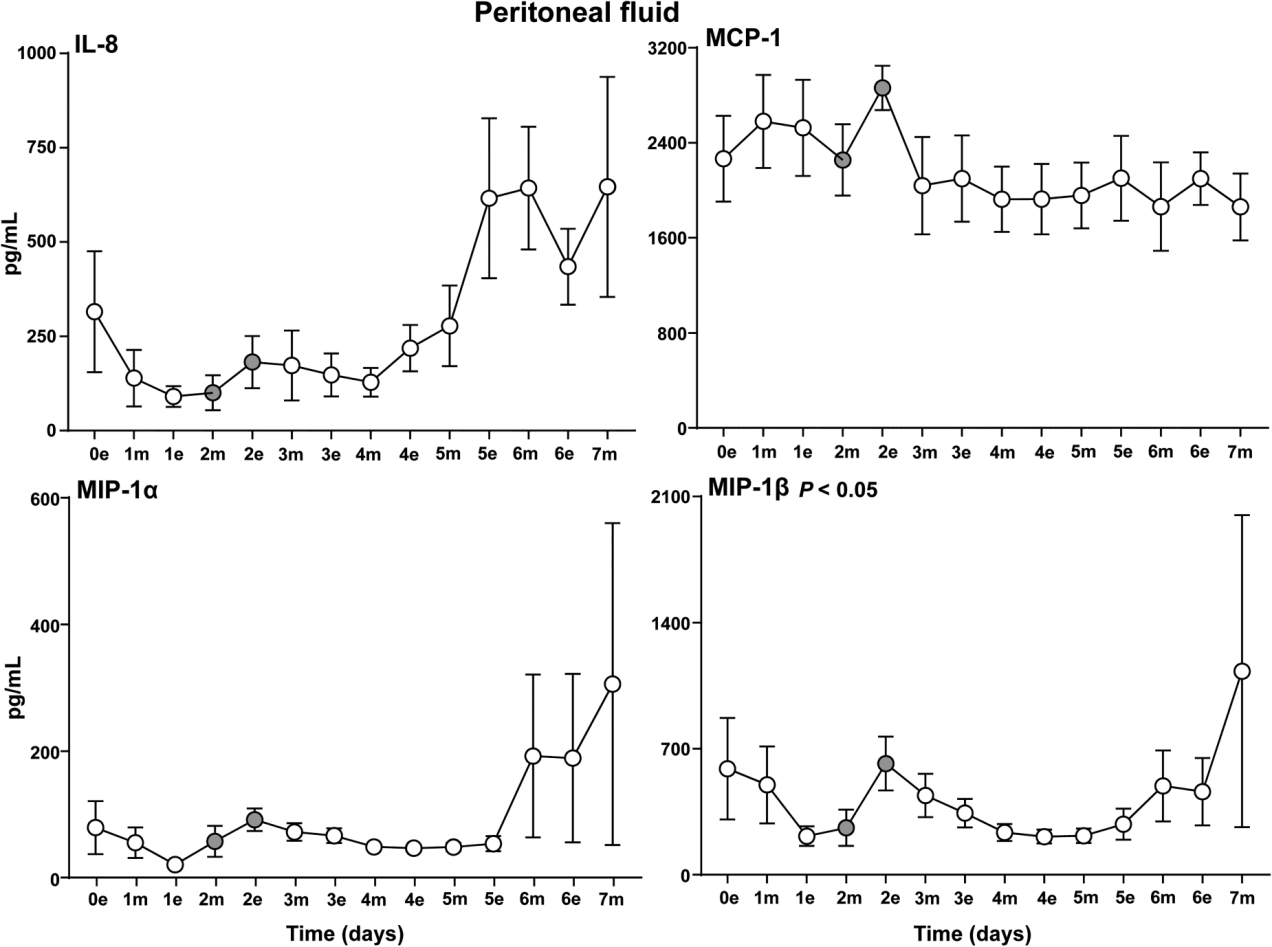
Chemokines in peritoneal fluid. Analysis of the chemokines IL-8, MCP-1, MIP-1α and MIP-1β in peritoneal fluid on the evening the day of surgery (0e) and the following seven postoperative days after CRS–HIPEC (*n* = 9). Intraperitoneal instillation of the α-emitting radionuclide therapeutic Radspherin® was conducted between time-point 2m and 2e (grey circles). Data are presented as mean and standard error of the mean. SCR = screening (baseline before surgery), 0 = day of operation, 1-7 = postoperative days. m = morning sample. e = evening sample. Grey circles = time-points before and after instillation of Radspherin®. *P*-values <0.05 were considered statistically significant.

The cytokine response could be divided into four different patterns. First, a high level immediately postoperatively and a subsequent gradual decline and stabilization afterwards, typically seen for the proinflammatory cytokines IL-6, IL-1β, IL-12 (Figure 3) and the anti-inflammatory cytokine IL-10 (Figure 4). Second, a striking pattern with a steep increase from postoperative day 1 to day 3 was typically seen for the proinflammatory cytokines INFγ, IP-10 and the anti-inflammatory cytokine IL-1RA (Figure 4). Third, a late substantial increase after 5 to 7 postoperative days was observed in particular for the proinflammatory cytokines TNF (Figure 3), IL8, MIP-1α, MIP-1β (Figure 5) and also to a lesser extent for G-CSF and FGF (Figure S5). Fourth, several cytokines had a relatively uneventful course after CRS–HIPEC, typically exemplified by MCP-1 (Figure 5), IL-7 and GM-CSF (Figure S5), and IL-2, IL-4 and IL-5 (Figure S6).

Four cytokines displayed a significant (*P *< 0.05) increase after IP administration of Radspherin®, TNF (Figure 3), IFNγ and IL1-RA (Figure 4), and MIP-1β (Figure 5).

### Urine Cytokines

In general, urine values were 1 to 25 folds lower than the corresponding intraperitoneal values (PF), and 12 of the 27 cytokines (IL-4, IL-12 p70, IL-13, IL-15, IL-17, MIP-1α, bFGF, G-CSF, GM-CSF, PDGF-BB, and VEGF) were below the detection limit at all time-points and were exclude from further analysis. However, an interesting pattern appeared for several of the cytokines, namely an abrupt increase the day of the procedure or the first post-operative day. This pattern was typically seen for the cytokines IL-6 and IL-RA (Figure 2), IFNγ (Figure S4), IL-10, MIP-1β and eotaxin (Figure S7) and to a lesser extent for IP-10 (Figure S4), IL-5 and IL-9 (Figure S8). The same abrupt increase was found for IL-6 and IL1-RA in plasma (Figure 2), but not for the other cytokines mentioned above.

No particular effect was seen after IP administration of Radspherin® for any of the cytokines detected in urine.

## Discussion

A compartmentalized inflammatory response with particular time-dependent differences and patterns was observed systemically (in plasma), locally in the peritoneal cavity (PF) and in urine after CRS–HIPEC. The α-emitting radionuclide therapeutic Radspherin® administered IP had an overall modest impact on the inflammation observed. Interestingly, the proinflammatory cytokines TNF and MIP-1β increased significantly in both plasma and PV immediately after instillation of Radspherin®.

The study is in line with the previous observation that despite major surgery, the systemic inflammatory response in plasma was relatively modest both in the number of cytokines detected to increase, and in their magnitude to increase, compared to the broad local intraperitoneal response.^6^ The time-dependent pattern of response was elaborated further compared to this previous study, as the time frame was expanded to 7 days postoperatively. Interestingly, a new pattern of inflammatory response was detected intraperitoneally by this prolonged observation period. A late wave of increased cytokines included chemokines such as the complement dependent IL-8 and the MIPs,^12–14^ but also growth factors, perhaps depicting an initiating phase of repair and rebuilding of damaged tissue after surgery.^15,16^ In light of this, one might argue that the main features of the four “patterns” of cytokine response detected represent a logical development from acute reaction to injury towards repair after complex surgery.

First an immediate inflammatory reaction is seen, followed by a second wave after 1 to 3 days, with a third wave emerging towards the end of the time frame after 5 to 7 days. A forth group of cytokines displayed a relatively uneventful course. The immediate response with typical pro- and anti-inflammatory cytokines like IL-6 and IL-10, respectively, was detected in the first wave. Of the few studies published on the subject, the immediate inflammatory cytokines like IL-6, known to induce fever,^17^ is also previously shown to emerge early IP after abdominal surgery.^18,19^

The anti-inflammatory cytokine IL-10 exhibited the same pattern, balancing the inflammatory response. Interestingly, in the second wave chemokines like IP-10 increased substantially to a sustained high level through the observation period. IP-10 not only attracts immune cells but is also part of an appropriate remodeling of fibrosis and repair during wound healing.^20^ Even the second wave included anti-inflammatory counterparts such as the anti-inflammatory cytokine IL-1RA, not least balancing the induction of IL-1β in the first wave.^21^ Notably, the second wave included activation of the complement cascade. Interestingly, complement activation occurred a day earlier in plasma than in PV, most likely reflecting that plasma activation occurs immediately, and is delayed intraperitoneal. The mitomycin C (MMC)-based HIPEC could influence the production and release of complement proteins locally in the peritoneal cavity, as impact on the immune system is known to occur after the treatment.^22^ Finally, the adaptive cytokines displayed an uneventful course, reflecting that the adaptive immune system is not immediately activated after extensive surgery, but probably are gradually involved later on.^23^

Little is known about inflammation markers in urine after CRS–HIPEC. Studies are published on acute kidney injury after CRS and nephrotoxic cisplatin-based HIPEC,^24,25^ with probably a range of proteins passing the glomeruli to urine depending on their molecular weight. None of the patients in the present study with mitomycin-C-based HIPEC, experienced kidney failure or exhibited reduced renal function in blood samples. Despite this, abrupt increased levels of total protein count and in particular low molecular weight cytokines like IL-6 and IL1-RA was detected in urine immediately after surgery. The rise of cytokines in urine seems to be caused by a glomerular leak from plasma and not de novo production and release of the cytokines in the urinary tract, as the increased plasma levels of IL-6 and IL1-RA corresponded with the rise in urine. Not all low molecular weight cytokines *eg* TNF increased in urine. It is known from in vitro studies of *eg* catheters with molecular weight cut-off filters that many cytokines with substantially lower molecular weight than the cut-off will not pass,^26^ as shape including folding and charge of the proteins, among other factors, influence passage through biological and artificial filters.

Radspherin® had modest effects on the immune responses systemically in plasma, as well as locally in the peritoneal cavity and in urine, with a few interesting exceptions. TNF and MIP-1β increased significantly both in plasma and PF, IL-9 only in plasma and IFNγ and IL1-RA only in PF after instillation of Radspherin®. It is known that the innate immune system is triggered by artificial surfaces of various origins,^27,28^ and that the cytokine response may differ considerably depending on the molecular structure of the surface.^29^ The biodegradable calcium carbonate microparticles on which the α-emitting radionuclide radium-224 (^224^Ra) is adsorbed, might have acted as an artificial surface with a limited immune activating ability. However, the results must be interpreted with caution, as the number of cases and numbers of inflammatory markers induced are limited. For the vast majority of the cytokines as well as complement activation products, a non-significant minor increase or decrease was seen which overall may be interpreted as Radspherin® had little effect on the overall inflammatory response.

Inflammation is a double edge sword in terms of cancer cell elimination. On one side, a low grade long lasting inflammation in the microenvironment surrounding the tumor might trigger and nourish tumor development and is regarded as one of the hallmarks of cancer.^30^ On the other hand increased levels of innate proinflammatory cytokines, such as TNF, IL-6, IL-1β and the strong T-cell stimulator IFNγ are all associated with immunological cell death,^31^ which might be beneficial to long-term disease-free and overall survival.^6^ When an artificial surface is implemented in the body, proteins accumulate around the object creating a new interface with induction of cascades like the complement and the coagulation systems,^32^ followed by a broader immune activation with a range of cytokines and recruitment of immune cells. If a drug like Radspherin® had been incompatible and immunogenic, it would probably have been contra productive as the short-range α-emission would have been blocked by the protein film created.^33^ Despite the general potential synergetic effects of innate immediate immune activation for cancer cell killing, it is therefore probably beneficial for Radspherin® to be inert.

The main limitations of this study are related to the descriptive nature of the data, a limited number of patients and the lack of a control group with CRS–HIPEC patients without Radspherin® instillation. The results obtained in this exploratory study give indications in particular from the time points before and after instillation, that Radspherin® may have limited impact on inflammation. Despite this, one cannot exclude that *eg* the late wave of growth factor increase interpreted as an initiating phase of repair and rebuilding of damaged tissue after surgery, may have something to do with Radspherin®. Thus, future studies should include a control group. Further, CRS is by nature heterogeneous as peritoneal metastasis spread differs from patient to patient and thus will peritonectomy procedures differ as well. Although the study is exploratory and causality cannot be convincingly concluded, the results may form an important basis for future studies and have establish the inflammatory, or lack of such, potential for Radspherin®.

In conclusion, a substantial inflammatory response was observed in the peritoneal cavity after CRS–HIPEC for PM-CRC with a prolonged phase dominated by cytokines involved in rebuilding and repair. The systemic response in plasma was markedly lower. An early leak of selected cytokines through the glomeruli to urine was detected after CRS–HIPEC without resulting in kidney failure. Radspherin® administered IP had minor impact on the overall inflammatory response, which is probably beneficial given its mode of action.

## Supplemental Material

sj-tif-1-tct-10.1177_15330338231192902 - Supplemental material for Effect of Intraperitoneal ^224^Radium-Labelled Microparticles on Compartmentalized Inflammation After Cytoreductive Surgery and Hypertherm Intraperitoneal Chemotherapy Click here for additional data file. Supplemental material, sj-tif-1-tct-10.1177_15330338231192902 for Effect of Intraperitoneal ^224^Radium-Labelled Microparticles on Compartmentalized Inflammation After Cytoreductive Surgery and Hypertherm Intraperitoneal Chemotherapy by Ebbe Billmann Thorgersen, Jørund Asvall, Camilla Schjalm, Karin Ekholt McAdam, Øyvind Sverre Bruland, Stein Gunnar Larsen and Tom Eirik Mollnes in Technology in Cancer Research & Treatment

sj-tif-2-tct-10.1177_15330338231192902 - Supplemental material for Effect of Intraperitoneal ^224^Radium-Labelled Microparticles on Compartmentalized Inflammation After Cytoreductive Surgery and Hypertherm Intraperitoneal Chemotherapy Click here for additional data file. Supplemental material, sj-tif-2-tct-10.1177_15330338231192902 for Effect of Intraperitoneal ^224^Radium-Labelled Microparticles on Compartmentalized Inflammation After Cytoreductive Surgery and Hypertherm Intraperitoneal Chemotherapy by Ebbe Billmann Thorgersen, Jørund Asvall, Camilla Schjalm, Karin Ekholt McAdam, Øyvind Sverre Bruland, Stein Gunnar Larsen and Tom Eirik Mollnes in Technology in Cancer Research & Treatment

sj-tif-3-tct-10.1177_15330338231192902 - Supplemental material for Effect of Intraperitoneal ^224^Radium-Labelled Microparticles on Compartmentalized Inflammation After Cytoreductive Surgery and Hypertherm Intraperitoneal Chemotherapy Click here for additional data file. Supplemental material, sj-tif-3-tct-10.1177_15330338231192902 for Effect of Intraperitoneal ^224^Radium-Labelled Microparticles on Compartmentalized Inflammation After Cytoreductive Surgery and Hypertherm Intraperitoneal Chemotherapy by Ebbe Billmann Thorgersen, Jørund Asvall, Camilla Schjalm, Karin Ekholt McAdam, Øyvind Sverre Bruland, Stein Gunnar Larsen and Tom Eirik Mollnes in Technology in Cancer Research & Treatment

sj-tif-4-tct-10.1177_15330338231192902 - Supplemental material for Effect of Intraperitoneal ^224^Radium-Labelled Microparticles on Compartmentalized Inflammation After Cytoreductive Surgery and Hypertherm Intraperitoneal Chemotherapy Click here for additional data file. Supplemental material, sj-tif-4-tct-10.1177_15330338231192902 for Effect of Intraperitoneal ^224^Radium-Labelled Microparticles on Compartmentalized Inflammation After Cytoreductive Surgery and Hypertherm Intraperitoneal Chemotherapy by Ebbe Billmann Thorgersen, Jørund Asvall, Camilla Schjalm, Karin Ekholt McAdam, Øyvind Sverre Bruland, Stein Gunnar Larsen and Tom Eirik Mollnes in Technology in Cancer Research & Treatment

sj-tif-5-tct-10.1177_15330338231192902 - Supplemental material for Effect of Intraperitoneal ^224^Radium-Labelled Microparticles on Compartmentalized Inflammation After Cytoreductive Surgery and Hypertherm Intraperitoneal Chemotherapy Click here for additional data file. Supplemental material, sj-tif-5-tct-10.1177_15330338231192902 for Effect of Intraperitoneal ^224^Radium-Labelled Microparticles on Compartmentalized Inflammation After Cytoreductive Surgery and Hypertherm Intraperitoneal Chemotherapy by Ebbe Billmann Thorgersen, Jørund Asvall, Camilla Schjalm, Karin Ekholt McAdam, Øyvind Sverre Bruland, Stein Gunnar Larsen and Tom Eirik Mollnes in Technology in Cancer Research & Treatment

sj-tif-6-tct-10.1177_15330338231192902 - Supplemental material for Effect of Intraperitoneal ^224^Radium-Labelled Microparticles on Compartmentalized Inflammation After Cytoreductive Surgery and Hypertherm Intraperitoneal Chemotherapy Click here for additional data file. Supplemental material, sj-tif-6-tct-10.1177_15330338231192902 for Effect of Intraperitoneal ^224^Radium-Labelled Microparticles on Compartmentalized Inflammation After Cytoreductive Surgery and Hypertherm Intraperitoneal Chemotherapy by Ebbe Billmann Thorgersen, Jørund Asvall, Camilla Schjalm, Karin Ekholt McAdam, Øyvind Sverre Bruland, Stein Gunnar Larsen and Tom Eirik Mollnes in Technology in Cancer Research & Treatment

sj-tif-7-tct-10.1177_15330338231192902 - Supplemental material for Effect of Intraperitoneal ^224^Radium-Labelled Microparticles on Compartmentalized Inflammation After Cytoreductive Surgery and Hypertherm Intraperitoneal Chemotherapy Click here for additional data file. Supplemental material, sj-tif-7-tct-10.1177_15330338231192902 for Effect of Intraperitoneal ^224^Radium-Labelled Microparticles on Compartmentalized Inflammation After Cytoreductive Surgery and Hypertherm Intraperitoneal Chemotherapy by Ebbe Billmann Thorgersen, Jørund Asvall, Camilla Schjalm, Karin Ekholt McAdam, Øyvind Sverre Bruland, Stein Gunnar Larsen and Tom Eirik Mollnes in Technology in Cancer Research & Treatment

sj-tif-8-tct-10.1177_15330338231192902 - Supplemental material for Effect of Intraperitoneal ^224^Radium-Labelled Microparticles on Compartmentalized Inflammation After Cytoreductive Surgery and Hypertherm Intraperitoneal Chemotherapy Click here for additional data file. Supplemental material, sj-tif-8-tct-10.1177_15330338231192902 for Effect of Intraperitoneal ^224^Radium-Labelled Microparticles on Compartmentalized Inflammation After Cytoreductive Surgery and Hypertherm Intraperitoneal Chemotherapy by Ebbe Billmann Thorgersen, Jørund Asvall, Camilla Schjalm, Karin Ekholt McAdam, Øyvind Sverre Bruland, Stein Gunnar Larsen and Tom Eirik Mollnes in Technology in Cancer Research & Treatment

## Abbreviations:

CRS: cytoreductive surgery
EDTA: ethylenediaminetetraacetic acid
ELISA: enzyme-linked immunosorbent assay
HIPEC: hypertherm intraperitoneal chemotherapy
IL: interleukine
IP: intraperitoneal
PF: peritoneal fluid
PM-CRC: peritoneal metastases from colorectal cancer
TCC: terminal complement complex
