# Supplementary figures and images for: Effect of Intraperitoneal 224Radium-Labelled Microparticles on Compartmentalized Inflammation After Cytoreductive Surgery and Hypertherm Intraperitoneal Chemotherapy
Source: Technol Cancer Res Treat. 2023 Aug 14;22:15330338231192902. doi: 10.1177/15330338231192902 (PMC10426314; doi:10.1177/15330338231192902)

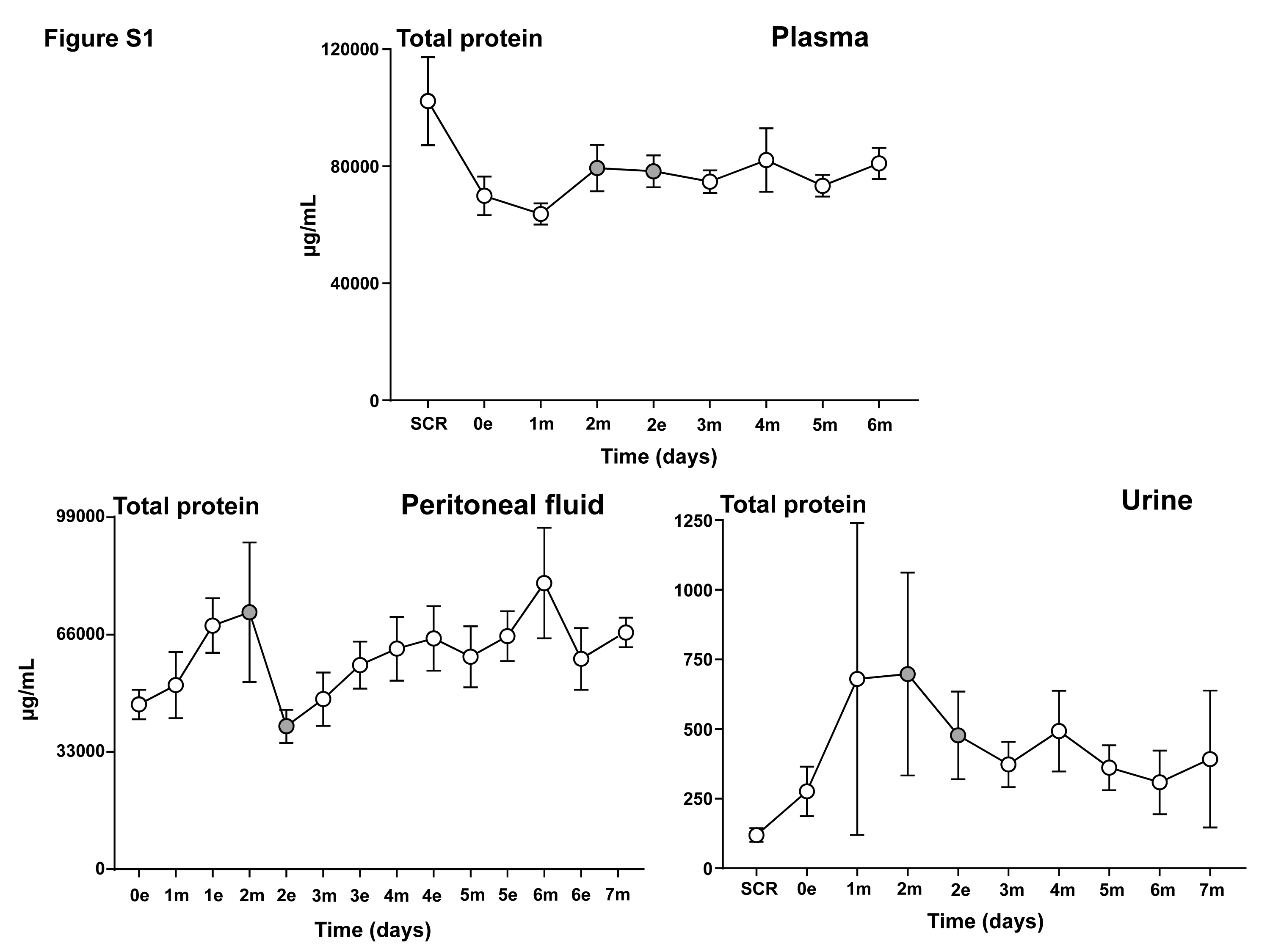

Supplement: sj-tif-1-tct-10.1177_15330338231192902 - Supplemental material for Effect of Intraperitoneal 224Radium-Labelled Microparticles on Compartmentalized Inflammation After Cytoreductive Surgery and Hypertherm Intraperitoneal Chemotherapy [file sj-tif-1-tct-10.1177_15330338231192902.tif]

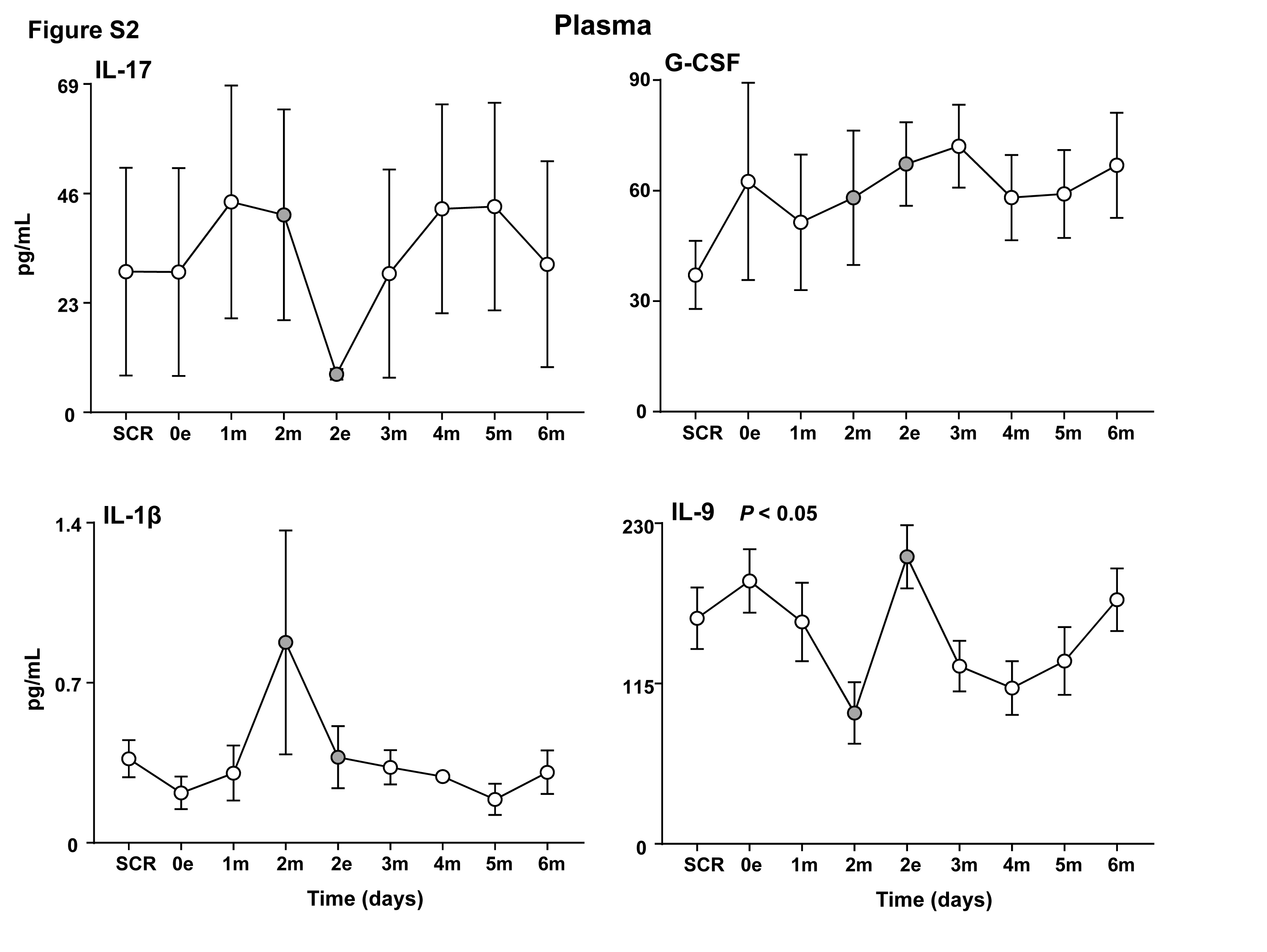

Supplement: sj-tif-2-tct-10.1177_15330338231192902 - Supplemental material for Effect of Intraperitoneal 224Radium-Labelled Microparticles on Compartmentalized Inflammation After Cytoreductive Surgery and Hypertherm Intraperitoneal Chemotherapy [file sj-tif-2-tct-10.1177_15330338231192902.tif]

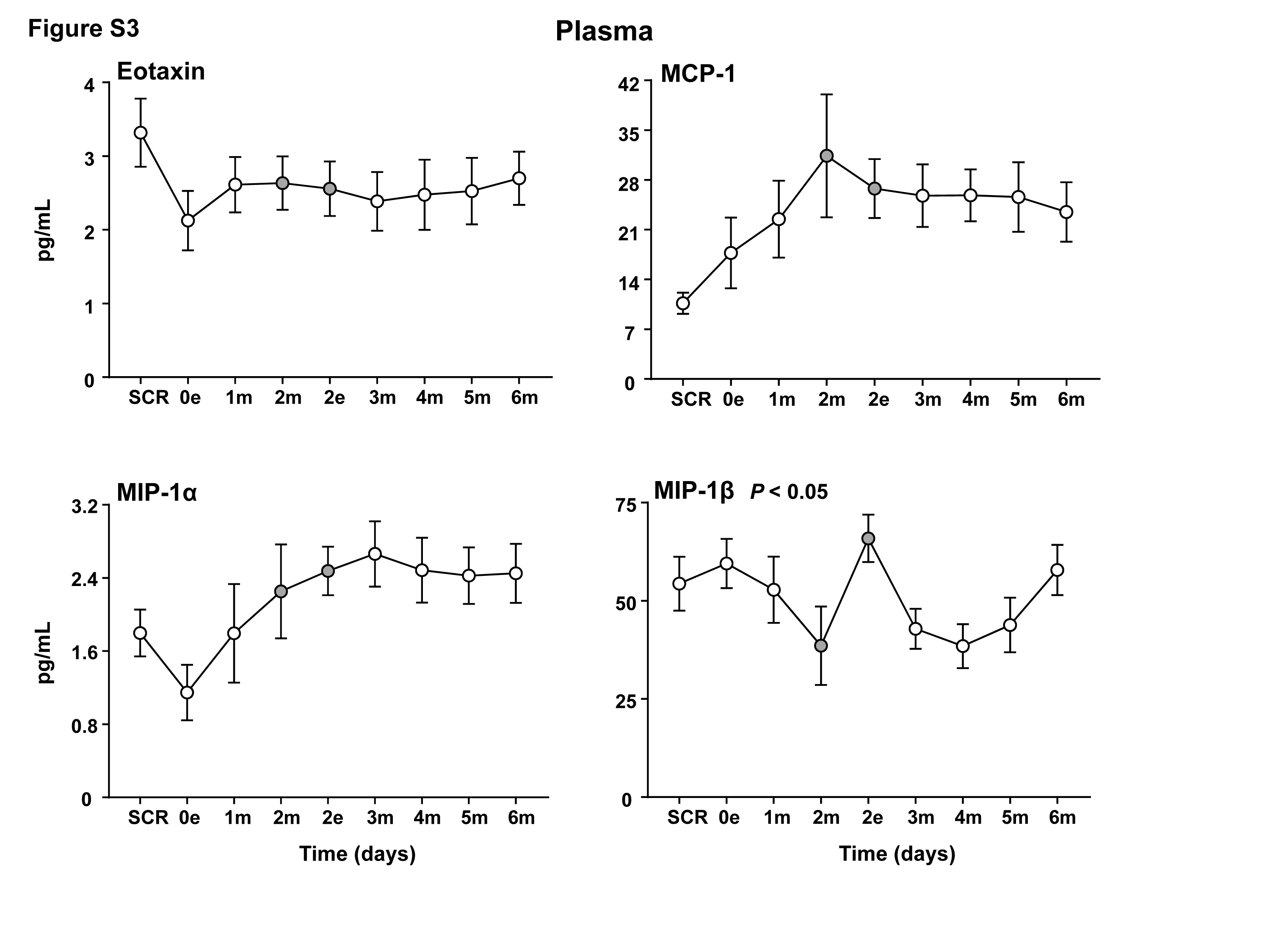

Supplement: sj-tif-3-tct-10.1177_15330338231192902 - Supplemental material for Effect of Intraperitoneal 224Radium-Labelled Microparticles on Compartmentalized Inflammation After Cytoreductive Surgery and Hypertherm Intraperitoneal Chemotherapy [file sj-tif-3-tct-10.1177_15330338231192902.tif]

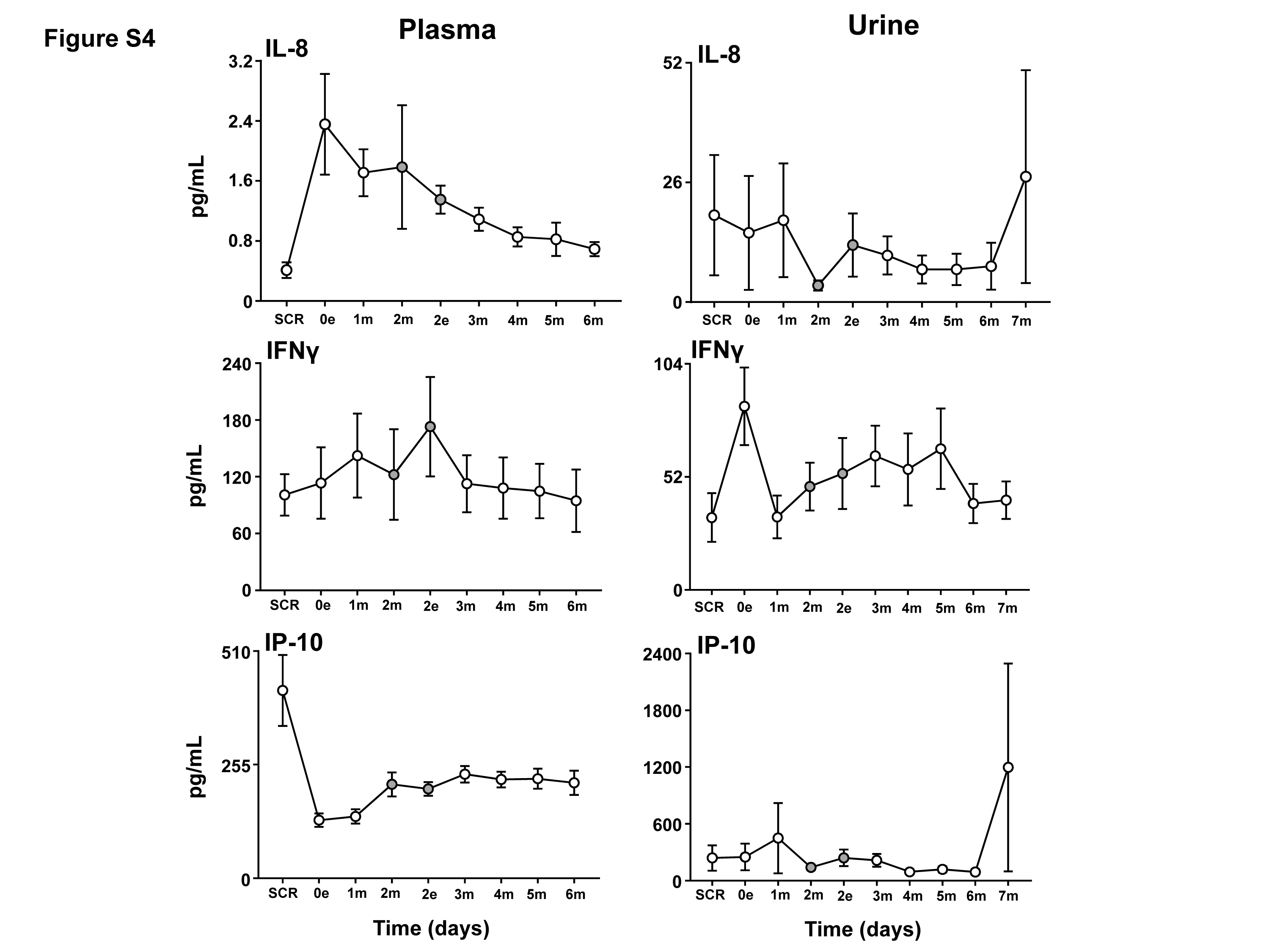

Supplement: sj-tif-4-tct-10.1177_15330338231192902 - Supplemental material for Effect of Intraperitoneal 224Radium-Labelled Microparticles on Compartmentalized Inflammation After Cytoreductive Surgery and Hypertherm Intraperitoneal Chemotherapy [file sj-tif-4-tct-10.1177_15330338231192902.tif]

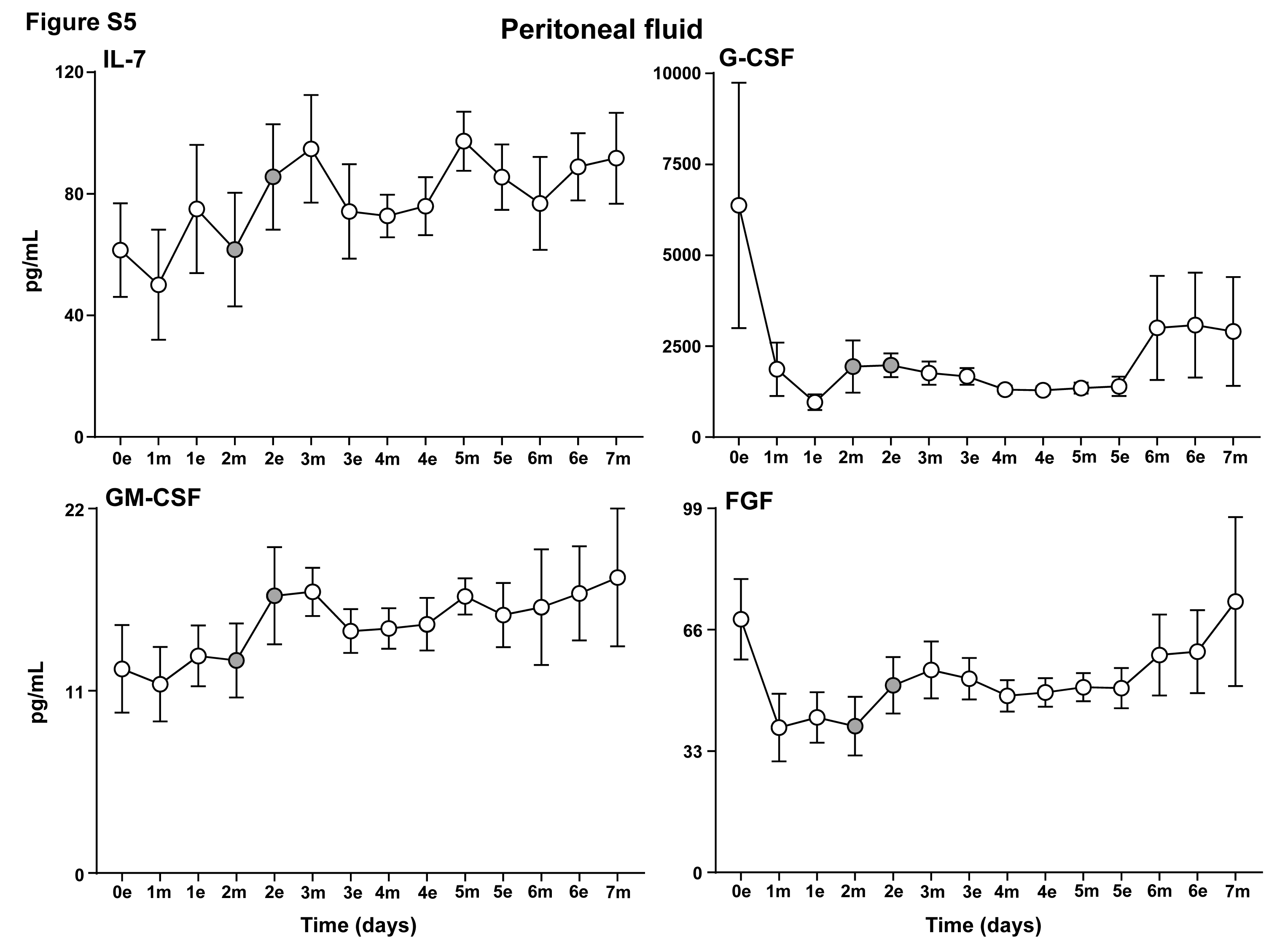

Supplement: sj-tif-5-tct-10.1177_15330338231192902 - Supplemental material for Effect of Intraperitoneal 224Radium-Labelled Microparticles on Compartmentalized Inflammation After Cytoreductive Surgery and Hypertherm Intraperitoneal Chemotherapy [file sj-tif-5-tct-10.1177_15330338231192902.tif]

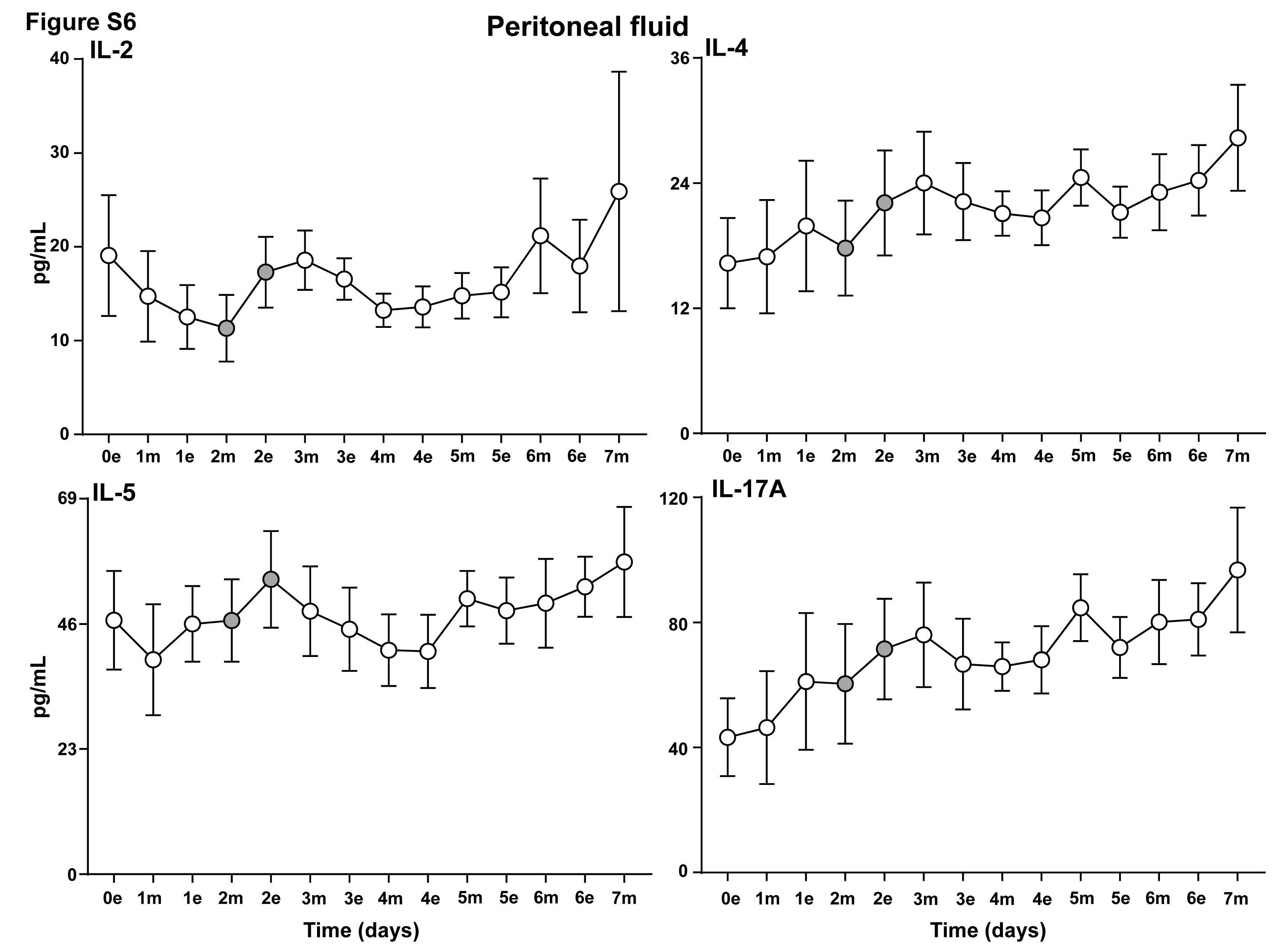

Supplement: sj-tif-6-tct-10.1177_15330338231192902 - Supplemental material for Effect of Intraperitoneal 224Radium-Labelled Microparticles on Compartmentalized Inflammation After Cytoreductive Surgery and Hypertherm Intraperitoneal Chemotherapy [file sj-tif-6-tct-10.1177_15330338231192902.tif]

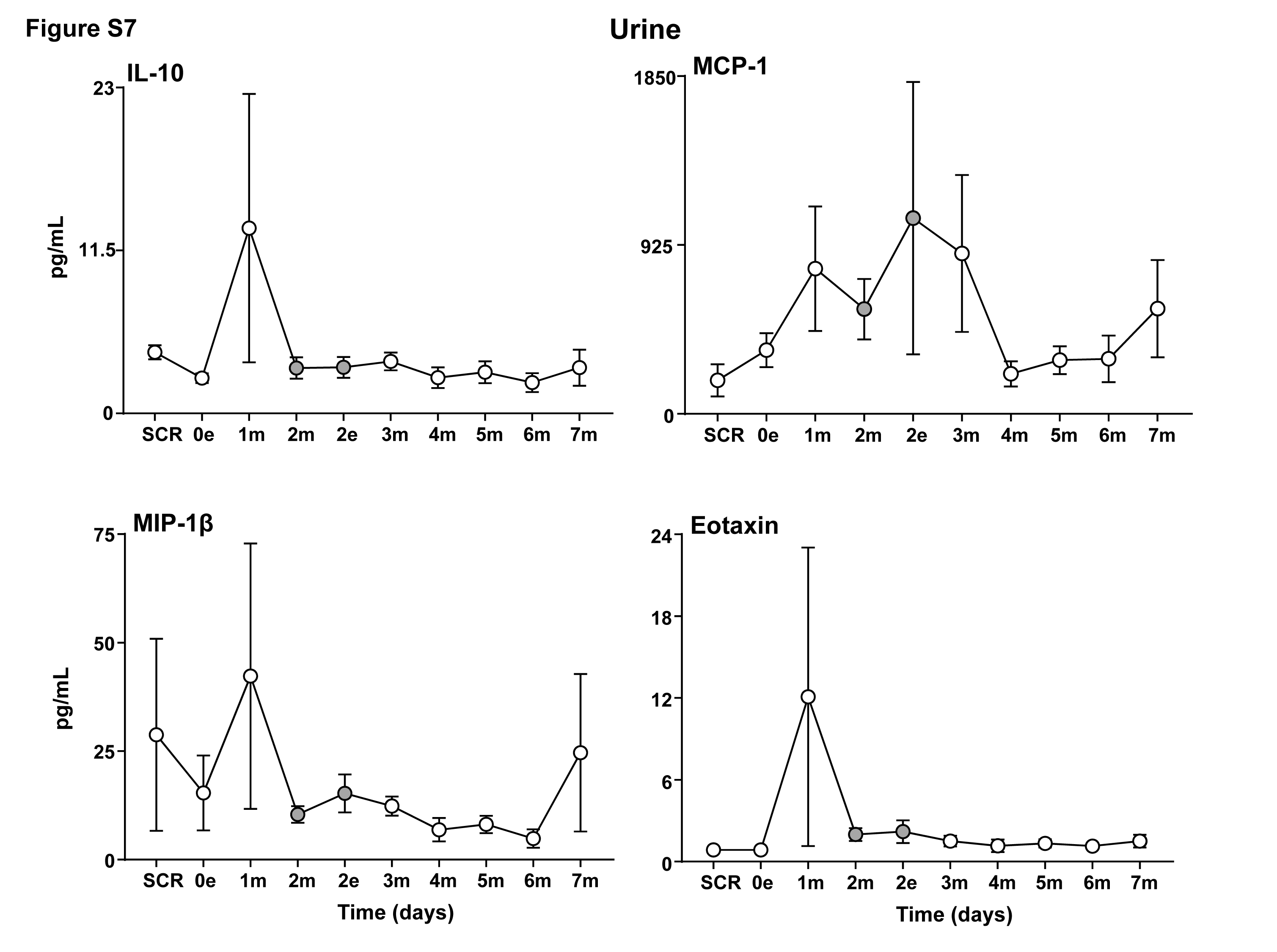

Supplement: sj-tif-7-tct-10.1177_15330338231192902 - Supplemental material for Effect of Intraperitoneal 224Radium-Labelled Microparticles on Compartmentalized Inflammation After Cytoreductive Surgery and Hypertherm Intraperitoneal Chemotherapy [file sj-tif-7-tct-10.1177_15330338231192902.tif]

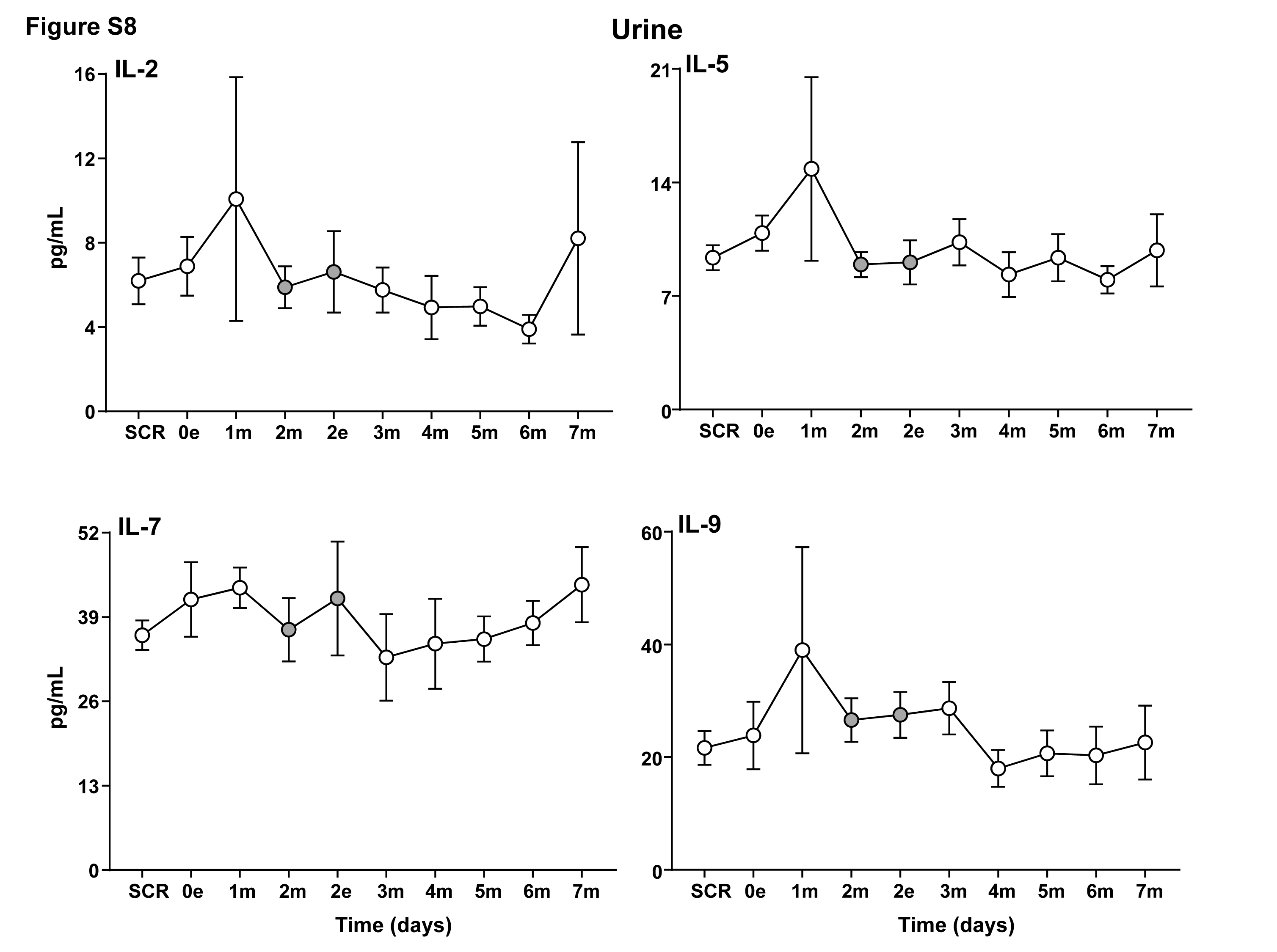

Supplement: sj-tif-8-tct-10.1177_15330338231192902 - Supplemental material for Effect of Intraperitoneal 224Radium-Labelled Microparticles on Compartmentalized Inflammation After Cytoreductive Surgery and Hypertherm Intraperitoneal Chemotherapy [file sj-tif-8-tct-10.1177_15330338231192902.tif]
